# Supplementary material for: The Effects of Human BDH2 on the Cell Cycle, Differentiation, and Apoptosis and Associations with Leukemia Transformation in Myelodysplastic Syndrome
Source: Int J Mol Sci. 2020 Apr 25;21(9):3033. doi: 10.3390/ijms21093033 (PMC7246807; doi:10.3390/ijms21093033)
Supplement: Supplementary file 1 [file ijms-21-03033-s001.pdf]

Supplement Methods

Cell Culture

The THP1, acute myelomonocytic leukemia cell line, was cultured in RPMI medium (GIBCO, Life Technologies, Carlsbad, CA, USA) supplemented with 10% fetal bovine serum (FBS), 1% streptomycin and penicillin (P/S), 4.5 g/L glucose, 10 mM HEPES ((4-(2-hydroxyethyl)-1-piperazineethanesulfonic acid),1 mM sodium pyruvate, and 1% β-mercaptoethanol. Puromycin (1 μg/mL) (Sigma, St. Louis, MO, USA) was added for stress selection of THP1 cells infected with shRNA empty vector and shRNA-BDH2 lentivirus. Approximately 1×10^5cells were plated in 10 cm dishes and cultured at 37°C in a humidified atmosphere containing 5% CO_2. Native cell line was purchased from the Food Industry Research and Development Institute, Taiwan, in 2012, May. The THP1 cell line was confirmed mycoplasma free but was not authenticated. However, we amplified cells when we received and used the initial stocked cells to do study.

mRNA Isolation and Expression

BM was obtained by sternal or posterior iliac bone aspiration and collected into heparin tubes containing 60 USP unit of sodium heparin. Total RNA was extracted from BM buffy coat samples of the enrolled patients and BDH2-KD THP1 cells using Trizol (Invitrogen, Life Technologies). The RNA input (2 μg) for cDNA

synthesis was determined by measuring OD_{260}. RNA was transcribed to first-strand cDNA by using TaqMan® High Capacity Reverse Transcription Kit (Applied Biosystems, Life Technologies), according to the manufacturer's instructions.

The cDNA sequences of *BDH2* and *LCN2* were evaluated, and the specific forward and reverse primers and TaqMan® probe were designed using Primer Express software version 1.5 (Applied Biosystems, Life Technologies). The TaqMan® MGB probe was synthesized and labeled with fluorescein amidite (FAM) fluorescent dye (Applied Biosystems, Life Technologies).

mRNA expression levels of *BDH2* and *LCN2* were analyzed by qRT-PCR using the following primer sets and probes.

BDH2: Forward primer 5′-TTCCAGCGTCAAAGGAGTTGT-3′,

reverse primer 5′-TTCCTGGGCACACACAGTTG-3′,

TaqMan® MGB probe 5′-ACAGATGTGTGTGTACAGCAC-3′,

LCN2: Forward primer 5′-GGTATGTGGTAGGCCTGGCA-3′,

reverse primer 5′-AACAGGACGGAGGTGACATTGT-3′,

TaqMan® MGB probe 5′-ACCCGCAAAAGATGTA-3′.

All the reactions were carried out in a 25μL final volume containing 200 ng of cDNA (as total input RNA), 400 nM of each primer, 200 nM of probe, and 12.5μL of 2X TaqMan®Universal PCR Master Mix (Applied Biosystems, Life Technologies).

qRT-PCR was performed in an ABI Villi 7 Sequence Detector (Applied Biosystems, Life Technologies), employing the following PCR cycling parameters: 95°C for 10 min, followed by 40 cycles at 95°C for 20 s, and 60°C for 1 min. The expression levels of the *BDH2* and *LCN2* genes were normalized to the internal control β-actin to obtain the relative threshold cycle (ΔC_T). The C_T values of β-actin were controlled between 18 and 22.

Western Blot Analysis

Cells were lysed in cold lysis buffer (triton glycerol cell lysis buffer containing 1/1000 volumes of phenyl-methyl-sulfonyl fluoride in 200 mM of 100 ×

ethanol stored at 4°C) with protease inhibitor cocktail (Amresco LLC, Solon, OH, USA). Cell lysates were separated by SDS-PAGE and transferred to a polyvinylidene fluoride (PVDF) membrane (PerkinElmer, Waltham, MA, USA). Further, the PVDF membrane was probed with primary antibodies specific for BDH (Sigma), and β-actin (Millipore Corporation, Billerica, MA, USA) and appropriate secondary antibodies were added. After incubation with antibodies, proteins were detected with an enhanced chemiluminescence kit (ECL; PerkinElmer).

Cell Cycle Analysis

THP1 cells were cultured in serum-free medium for 2 days to induce cell cycle arrest. Thereafter, the medium was supplemented with 0.2% FBS and 1% FBS for a day each, and 10% FBS was added to the culture medium to restart the cell cycle. Approximately 5×10^6cells were collected, washed with cold phosphate buffered saline (PBS), and re-suspended in 50 μLPBS. A total volume of 3mL of 70% cold ethanol was then added drop by drop. The samples were incubated at -20°C for 1 h and centrifuged at 300 × g for 5 min. The supernatant was removed, and the cell pellet was washed twice with 5mL cold PBS, re-suspended in 1mLprotease inhibitor (PI)/Triton X-100 (PI 20μg/mL; Triton-X 100 0.1%; RNase A 0.2 mg/mL) (PI from Beckman Coulter; Triton-X 100 and RNase A from Sigma), and incubated at room temperature for 30 min. Cell cycle was analyzed by flow cytometry.

Flow Cytometry and Cell Differentiation

Flowcytometry was performed using Gallios™ flowcytometer (BeckmanCoulter, USA). A total of 1×10^6 cells were collected, washed with cold PBS, re-suspended in 500 μL PBS, and double stained with 100 μL/mL CD markers for 20 min. THP1 cells were initiated to differentiate along the monocytic lineage following exposure to 1.25-dihydroxyvitaminD3 (Vit D3; Sigma) as described previously.[22] Approximately, 2.5×10^5 THP1 cells/mL were treated with 10^{-7} M Vit D3 in a

humidified atmosphere containing 5% CO_2, for 72 h at 37°C.Cell differentiation was detected by using the following antibodies: CD11b-PE and CD15-FITC, CD14-PE and CD64-FITC, CD16-PE and CD14-FITC double stain, and immunoglobulin G1-PE and immunoglobulin G1-FITC double stain (control markers) (Beckman Coulter).

Special stains

THP1 cells were prepared by cytospin after washing twice with PBS. For Liu stain, cells were stained with Liu A solution for 30 s and Liu B solution for 1 min. Cells were washed with water and observed under a microscope. For myeloperoxidase (MPO) staining, cells were fixed with 10% ethanol-formalin for 1 min, washed and dried, and then stained by POX staining solution for 30 s, followed by washing and drying and finally staining with Neutral Red for 30 s. For nonspecific esterase (NSE) staining, cells were fixed with buffered formol-acetone fixation buffer for 30 s. Staining solution was prepared by mixing 10 μL α-naphthyl butyrate, 500 μL ethylene glycol mono-methyl ether, and 100 μL solution of 4% pararosaniline HCL and 4% $NaNO_2$; the volume was made up to 10 mL by adding 9.4 mL PBS. Fixed cells were stained with staining solution for 45 min, and washed and dried again. Finally, the cells were stained with hematoxylin for 10 min, followed by washing and drying of

the stained cells.

Immunohistochemistry

For preparing slides, 5-μm thick sections from representative tissue blocks were cut, de-paraffinized with xylene, rinsed, and rehydrated with a graded alcohol series (100%, 95%, 85%, and 75%) for 5 min each, and then finally rinsed with distilled water. The slides were autoclaved in sodium citrate buffer (10 mM, pH 6.0) for 30 min to enhance antigen retrieval. Endogenous peroxidase activity was quenched by incubation in 3% H_2O_2/methanol buffer for 30 min. The slides were incubated with a monoclonal antibody against human BDH2 (sc-104197, Santa Cruz Biotechnology, Dallas, TX, USA) at a dilution of 1:200 overnight at 4°C in humidified chambers. These slides were washed three times in PBS and incubated with a biotinylated secondary antibody for 30 min at room temperature. Antigen-antibody complexes were detected by the avidin-biotin-peroxidase method using 3,3-diaminobenzidine as a chromogenic substrate (Dako, Glostrup, Denmark). Finally, the slides were counterstained with hematoxylin and then examined by light microscopy.

Figure S1.

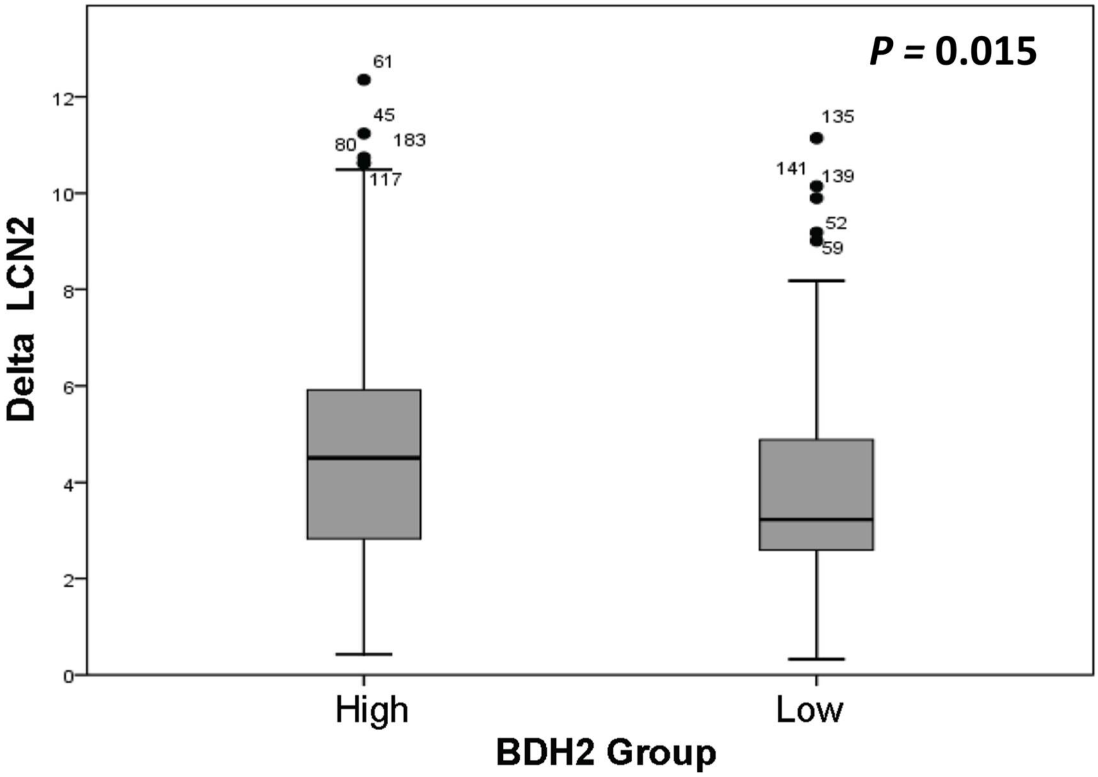

Figure S1. ***LCN2* mRNA expression in the bone marrow in high and low *BDH2* expression groups**. The expression levels *LCN2* were normalized to the internal control β-actin to obtain the relative threshold cycle (ΔC_T). BDH2, hydroxybutyrate dehydrogenase type 2; LCN2, lipocalin 2; qRT-PCR, quantitative reverse-transcription polymerase chain reaction.

Figure S2.

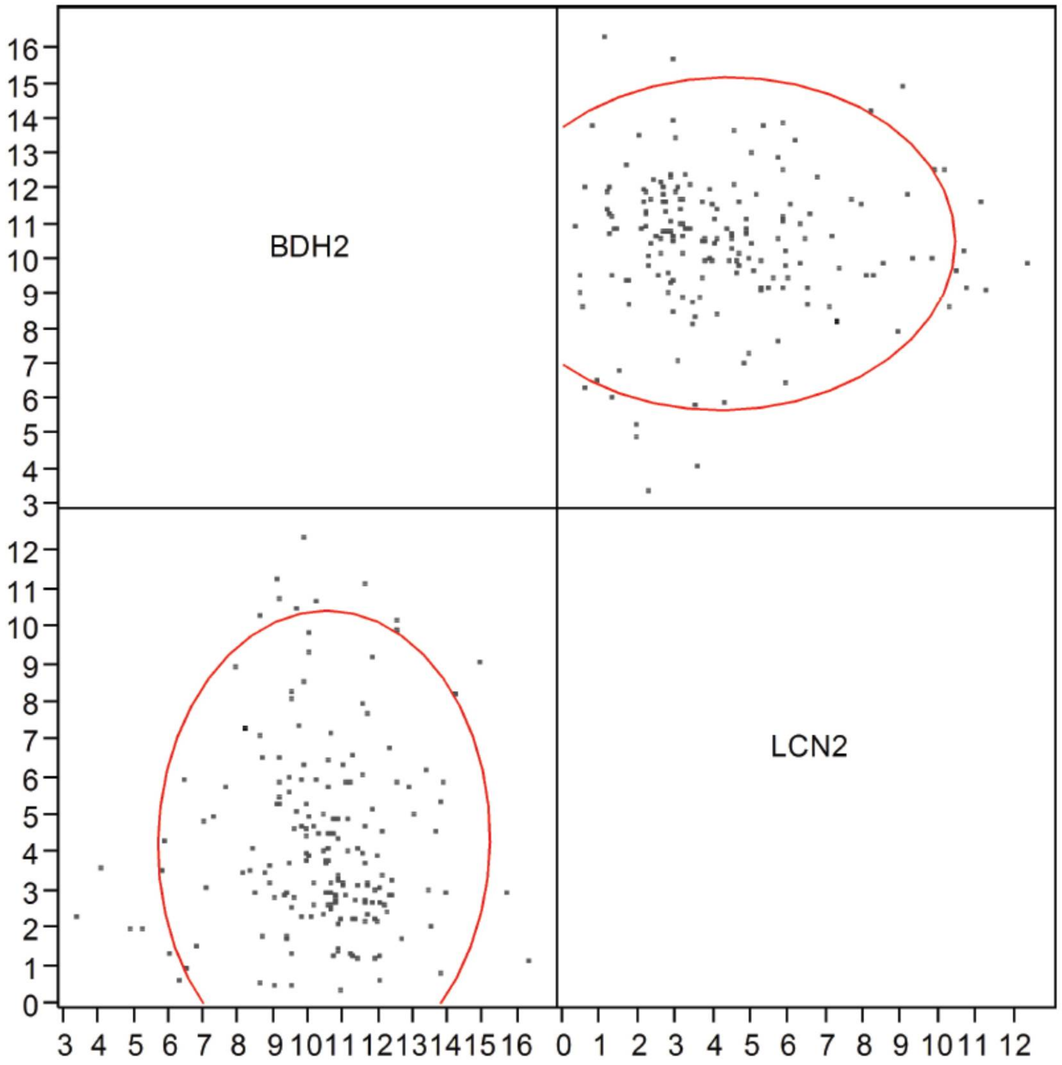

Pairwise correlation

Variable	by Variable	Correlation	Count	Lower 95%	Upper 95%	Signif Prob Plot Corr
LCN2	BDH2	0.0171	185	-0.1275	0.1610	0.8169

Figure S2. The correlation between *BDH2* and *LCN2* mRNA expression in bone marrow of MDS patients. There was no correlation between *BDH2* and *LCN2* mRNA expression in bone marrow of MDS patients. The expression levels of the *BDH2* and *LCN2* genes were normalized to the internal control β-actin to obtain the relative threshold cycle (ΔC_T). BDH2, hydroxybutyrate dehydrogenase type 2; LCN2, lipocalin 2.

Figure S3.

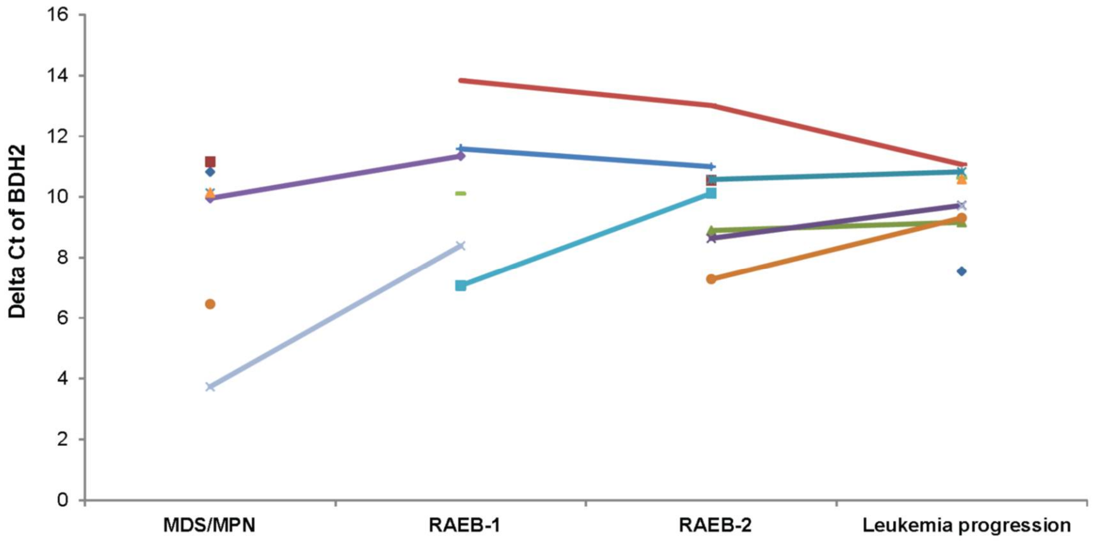

Figure S3. Expression of *BDH2* mRNA expression in 13 MDS patients with series change. BDH2, hydroxybutyrate dehydrogenase type 2; MDS, myelodysplastic syndrome; qRT-PCR, quantitative reverse-transcription polymerase chain reaction; RAEB, refractory anemia with excess blasts; RARS, refractory anemia with ringed sideroblasts.

Figure S4.

A

		ferritin	BDH2	LCN2
**ferritin**	Pearson Correlation	1	-.074	.111
	P Value		.221	.129
	Number	115	109	107
**BDH2**	Pearson Correlation	-.074	1	.017
	P Value	.221		.408
	Number	109	187	185
**LCN2**	Pearson Correlation	.111	.017	1
	P Value	.129	.408	
	Number	107	185	185

B

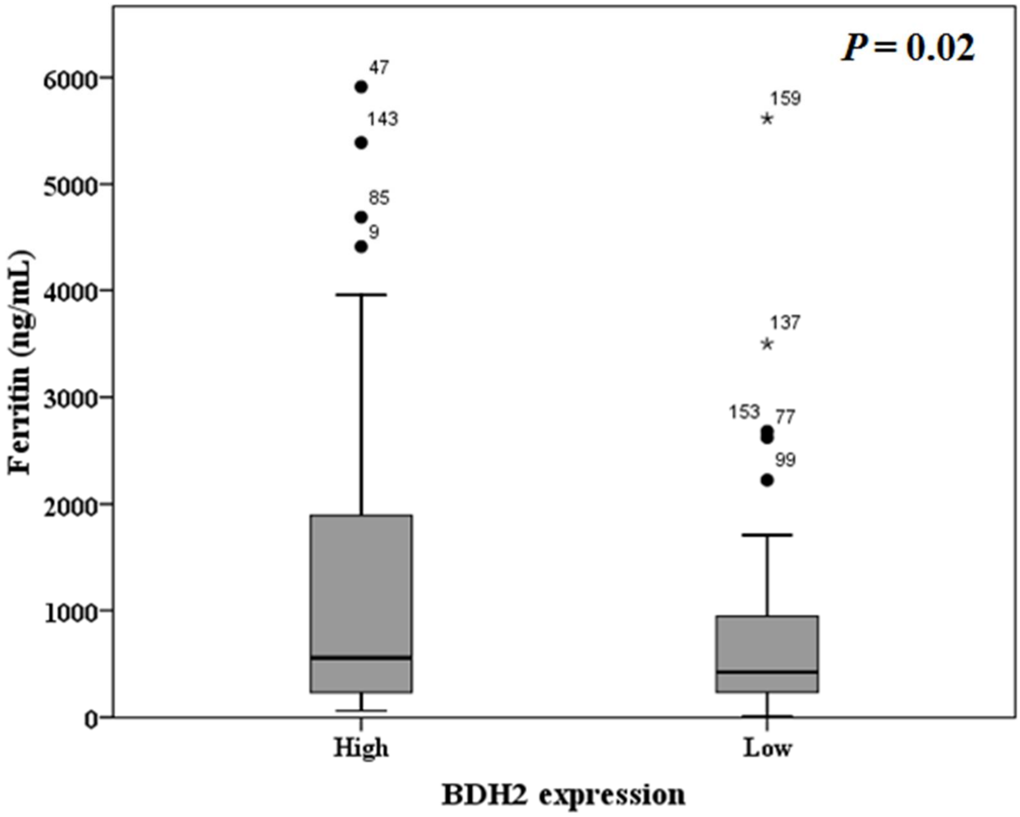

Figure S4. Correlation between serum ferritin levels and the expression levels of *BDH2* and *LCN2*. Ferritin levels were obtained for 115 patients who did not undergo repeated blood transfusion. (A) Correlation regression analysis was used to assess the correlation between *BDH2* and *LCN2* expression and *BDH2* and ferritin levels. (B) Ferritin levels were compared between the high and low *BDH2* expression groups**.** BDH2,hydroxybutyrate dehydrogenase type 2; LCN2, lipocalin 2.

Figure S5.

A

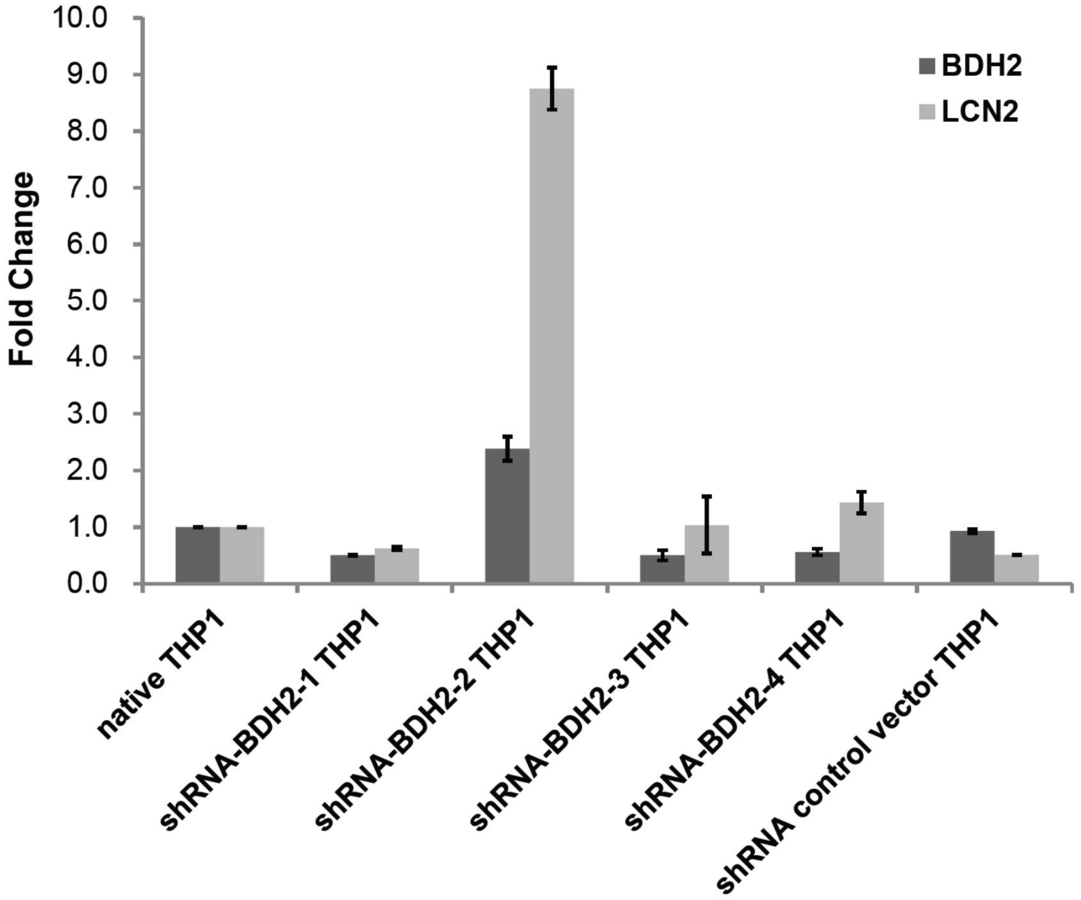

10.0
9.0
8.0
7.0
6.0
5.0
4.0
3.0
2.0
1.0
0.0
Fold Change
BDH2
LCN2
native THP1
shRNA-BDH2-1 THP1
shRNA-BDH2-2 THP1
shRNA-BDH2-3 THP1
shRNA-BDH2-4 THP1
shRNA control vector THP1

B

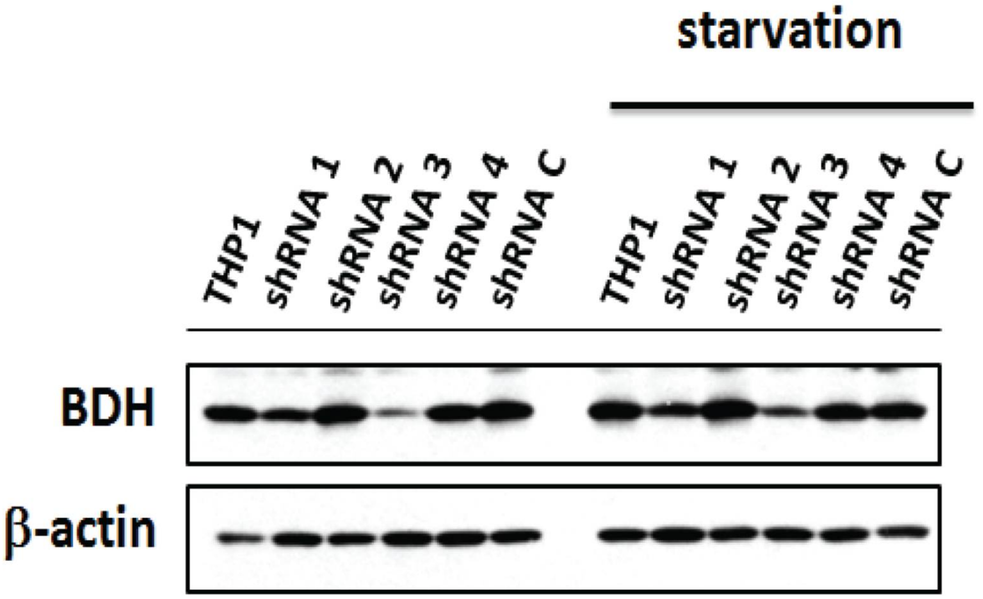

starvation
THP1
shRNA 1
shRNA 2
shRNA 3
shRNA 4
shRNA C
THP1
shRNA 1
shRNA 2
shRNA 3
shRNA 4
shRNA C
BDH
β-actin

Figure S5. The efficiency of shRNA interference of BDH2 in THP1 cells. BDH2-KD efficiency of four different clones, shRNA-BDH2-1, shRNA-BDH2-2, shRNA-BDH2-3, and shRNA-BDH2-4 was assessed by (A) quantitative reverse-transcription polymerase chain reaction and, (B) western blot analyses**.** The mRNA expression levels of the *BDH2* and *LCN2* genes were normalized to the internal control β-actin to obtain the relative threshold cycle (ΔC_T). For western blot analysis, antibodies specific for BDH and β-actin were used. BDH2 expression groups, respectively. BDH2, hydroxybutyrate dehydrogenase type 2; KD: knockdown; LCN2, lipocalin 2.

Figure S6.

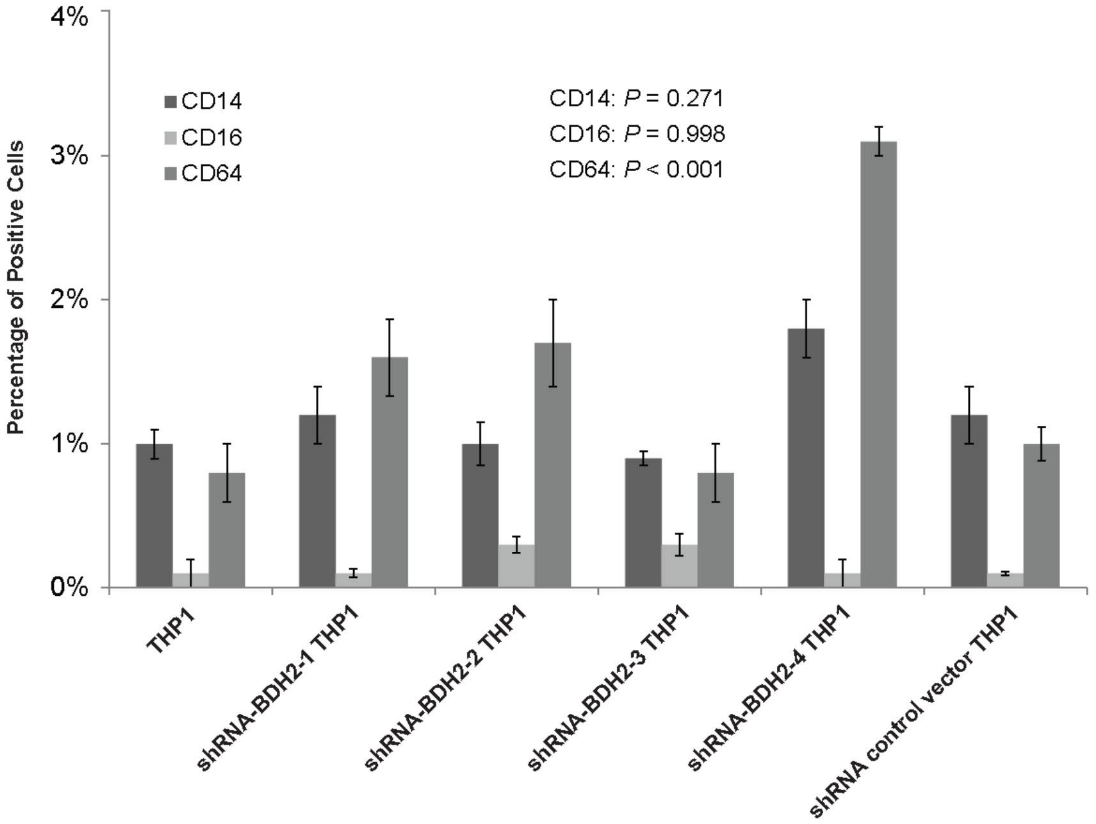

Figure S6. Surface markers indicate THP1 cells differentiation. CD14, CD16, and CD64 expression in THP1 and BDH2-KD THP1 cells, after treated with Vitamin D3 to induce differentiation. Only CD64 had significant difference between BDH2-KD and control THP1 cells. However, there was no correlation with BDH2 knockdown levels. BDH2, hydroxybutyrate dehydrogenase type 2.

Figure S7.

A

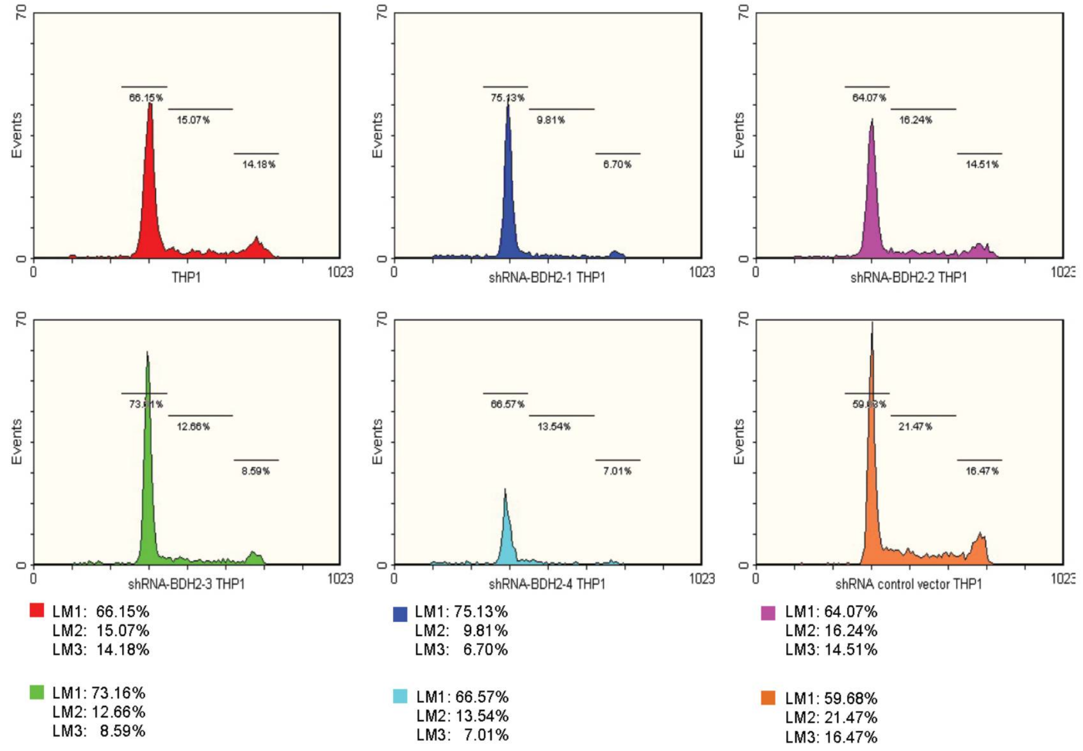

B

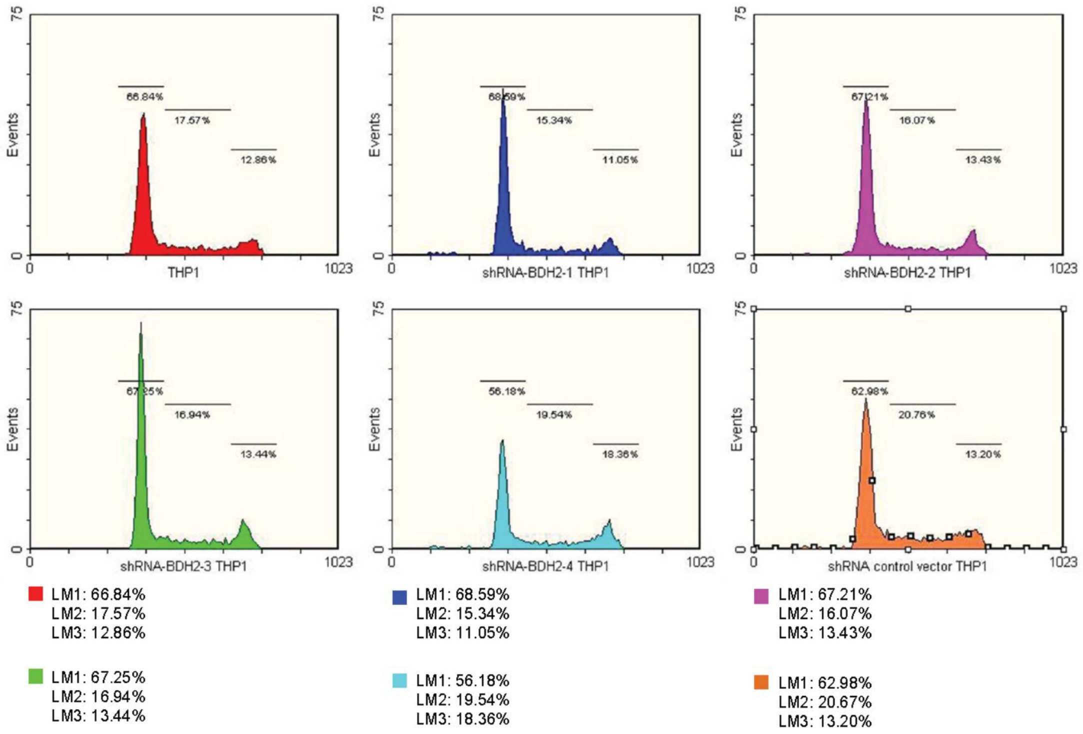

Figure S7. Cell cycle in shRNA control vector transfected THP1 cells. THP1 cells were cultured in serum-free medium for 2 days to induce cell cycle arrest. The

medium was supplemented with 0.2% FBS and 1% FBS for a day each. Ten percent FBS was added to the culture medium to restart the cell cycle. Cell cycle profile on the (A) third and, (B) fourth day after re-growth in serum-containing medium**.** FBS, fetal bovine serum.

Figure S8.

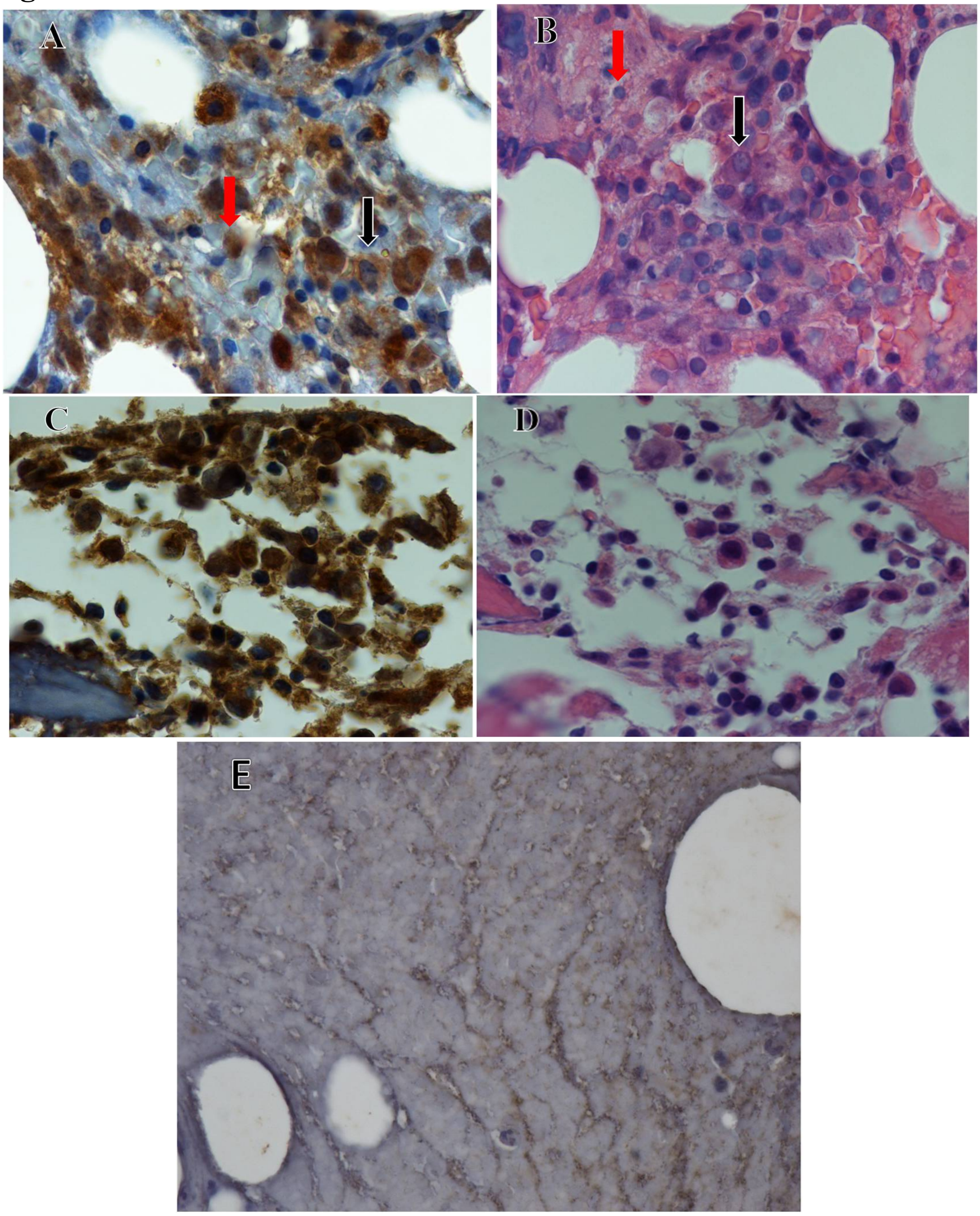

Figure S8. BDH2 protein expressions in the bone marrow. (A, C) The expression of BDH2 in the bone marrow of MDS patients was determined by immunohistochemistry using a monoclonal antibody specific for human BDH2. **(**B, D) The slides were counterstained with hematoxylin. The panels A and B were from

patients with high, and panels C and D were from patients with low expression of BDH2 in the bone marrow. (E) Control slide without using BDH2 antibody. The red arrows indicate normoblasts, which belong to erythroid series. The black arrows show myeloblasts.400x. BDH2, hydroxybutyrate dehydrogenase type 2.

Figure S9

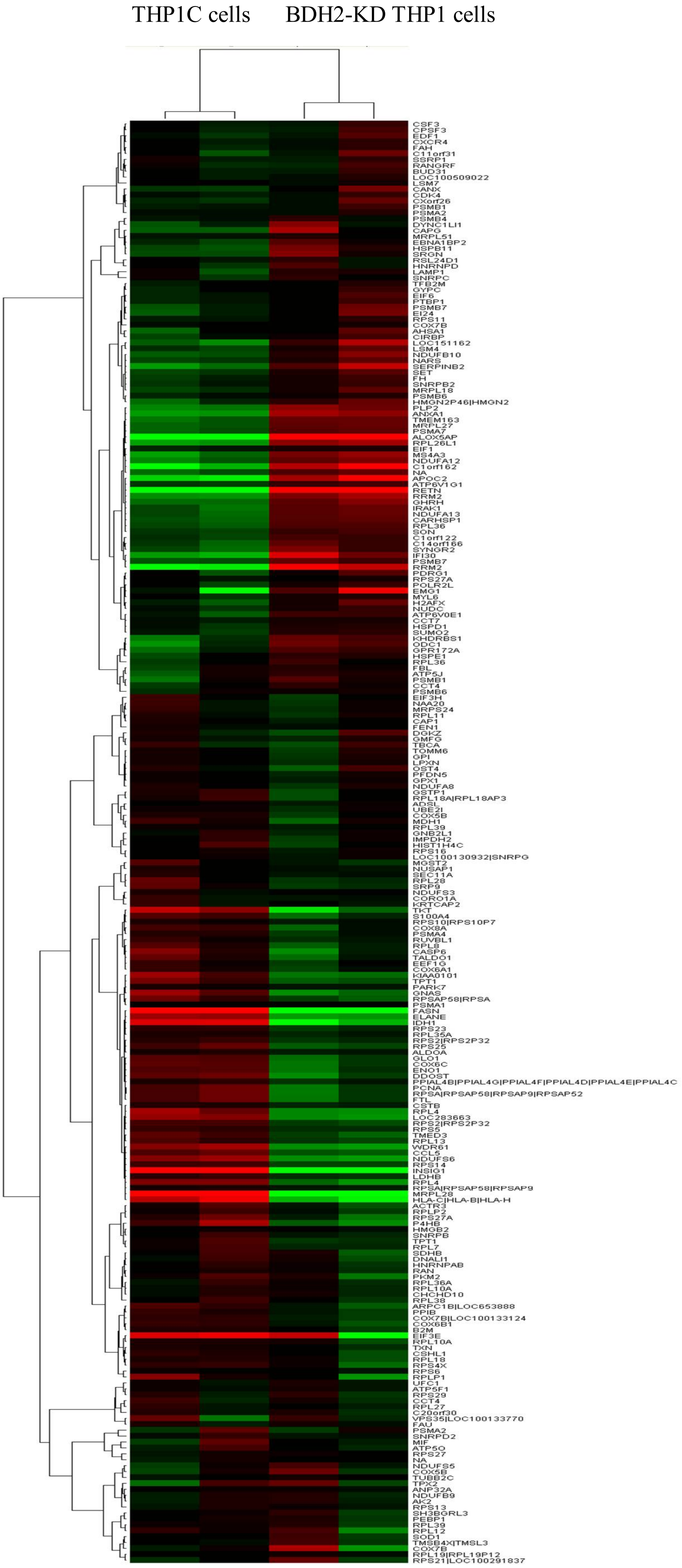

Figure S9. RNA microarray analysis between shRNA control vector transfected THP1 (THP1C, left two columns) and BDH2-KD THP1 cells (right two columns).

Supplement Table 1. Microarray analysis of BDH2-KD THP1 cells

**Gene symbol**	**ID**	**Discription**	**Log2 (T/C)#**	***P value***
NNT	PH_hs_0022275	nicotinamide nucleotide transhydrogenase	-1.12396	2.43E-10*
SLC25A1	PH_hs_0031529	solute carrier family 25 (mitochondrial carrier; citrate transporter), member 1	-1.16844	0.000258*
GBAS	PH_hs_0009186	glioblastoma amplified sequence	-1.04885	0.000001*
POLG	PH_hs_0023529	polymerase (DNA directed), gamma	-1.02335	0.000077*
TGFBR1	PH_hs_0022072	transforming growth factor, beta receptor I (activin A receptor type II-like kinase, 53kDa)	1.238682	0.000391*
SMAD3	PH_hs_0039929	SMAD, mothers against DPP homolog 3 (Drosophila)	-1.145608	0.000305*
PIK3CD	PH_hs_0033291	phosphoinositide-3-kinase, catalytic, delta polypeptide	-1.06438	0*
CAB39L	PH_hs_0048501	calcium binding protein 39-like	-1.732699	9.12E-13*
CREB3L2	PH_hs_0022159	cAMP responsive element binding protein 3-like 2	-1.073542	0.00007*
MAPK9	PH_hs_0045819	mitogen-activated protein kinase 9	1.177353	0.000016*
PRL	PH_hs_0035318	prolactin	1.572641	1.64E-13*
TP53INP2	PH_hs_0001086	tumor protein p53 inducible	-1.58863408925472	2.36E-10*
CCL20	PH_hs_0004333	chemokine (C-C motif) ligand 20	1.599742	6.64E-10*
IL8	PH_hs_0000069	interleukin 8	1.462731	2.17E-08*
TNFSF13B	PH_hs_0013929	tumor necrosis factor (ligand) superfamily, member 13b	-1.104741	3.51E-08*

MMP9	PH_hs_0009143	matrix metallopeptidase 9	-2.101386	1.34E-10*
CXCR2	PH_hs_0000102	chemokine (C-X-C motif) receptor 2	0.877452	0.021231

#T: BDH2-KD THP1 cells; C: control vector transfection THP1 cells

***statistics significant; *P* < 0.01**
